# Mechanistic definition of the cardiovascular mPGES-1/COX-2/ADMA axis

**DOI:** 10.1093/cvr/cvz290

**Published:** 2019-11-05

**Authors:** Nicholas S Kirkby, Joan Raouf, Blerina Ahmetaj-Shala, Bin Liu, Sarah I Mazi, Matthew L Edin, Mark Geoffrey Chambers, Marina Korotkova, Xiaomeng Wang, Walter Wahli, Darryl C Zeldin, Rolf Nüsing, Yingbi Zhou, Per-Johan Jakobsson, Jane A Mitchell

**Affiliations:** 1 National Heart & Lung Institute, Imperial College London, Dovehouse Street, London SW3 6LY, UK; 2 Unit of Rheumatology, Department of Medicine, Karolinska Institute, Stockholm, Sweden; 3 Cardiovascular Research Centre, Shantou University Medical College, Shantou, China; 4 King Fahad Cardiac Center, King Saud University, Riyadh, Saudi Arabia; 5 National Institute for Environmental Health Sciences, Durham, NC, USA; 6 Biotechnology and Autoimmunity Research, Eli Lilly and Company, Indianapolis, IN, USA; 7 Lee Kong Chian School of Medicine, Nanyang Technological University Singapore, Singapore, Singapore; 8 Institute of Molecular and Cell Biology, Agency for Science Technology & Research, Singapore, Singapore; 9 Department of Cell Biology, Institute of Ophthalmology, University College London, London, UK; 10 Singapore Eye Research Institute, Singapore, Singapore; 11 Center for Integrative Genomics, University of Lausanne, Lausanne, Switzerland; 12 Clinical Pharmacology and Pharmacotherapy Department, Goethe University, Frankfurt, Germany; 13 Karolinska University Hospital, Stockholm, Sweden

**Keywords:** Vioxx, Non-steroidal anti-inflammatory drugs, Methylarginines, ADMA, COX-2, Prostacyclin, PGE2

## Abstract

**Aims:**

Cardiovascular side effects caused by non-steroidal anti-inflammatory drugs (NSAIDs), which all inhibit cyclooxygenase (COX)-2, have prevented development of new drugs that target prostaglandins to treat inflammation and cancer. Microsomal prostaglandin E synthase-1 (mPGES-1) inhibitors have efficacy in the NSAID arena but their cardiovascular safety is not known. Our previous work identified asymmetric dimethylarginine (ADMA), an inhibitor of endothelial nitric oxide synthase, as a potential biomarker of cardiovascular toxicity associated with blockade of COX-2. Here, we have used pharmacological tools and genetically modified mice to delineate mPGES-1 and COX-2 in the regulation of ADMA.

**Methods and results:**

Inhibition of COX-2 but not mPGES-1 deletion resulted in increased plasma ADMA levels. mPGES-1 deletion but not COX-2 inhibition resulted in increased plasma prostacyclin levels. These differences were explained by distinct compartmentalization of COX-2 and mPGES-1 in the kidney. Data from prostanoid synthase/receptor knockout mice showed that the COX-2/ADMA axis is controlled by prostacyclin receptors (IP and PPARβ/δ) and the inhibitory PGE_2_ receptor EP4, but not other PGE_2_ receptors.

**Conclusion:**

These data demonstrate that inhibition of mPGES-1 spares the renal COX-2/ADMA pathway and define mechanistically how COX-2 regulates ADMA.

## Graphical Abstract

**Graphical Abstract:**
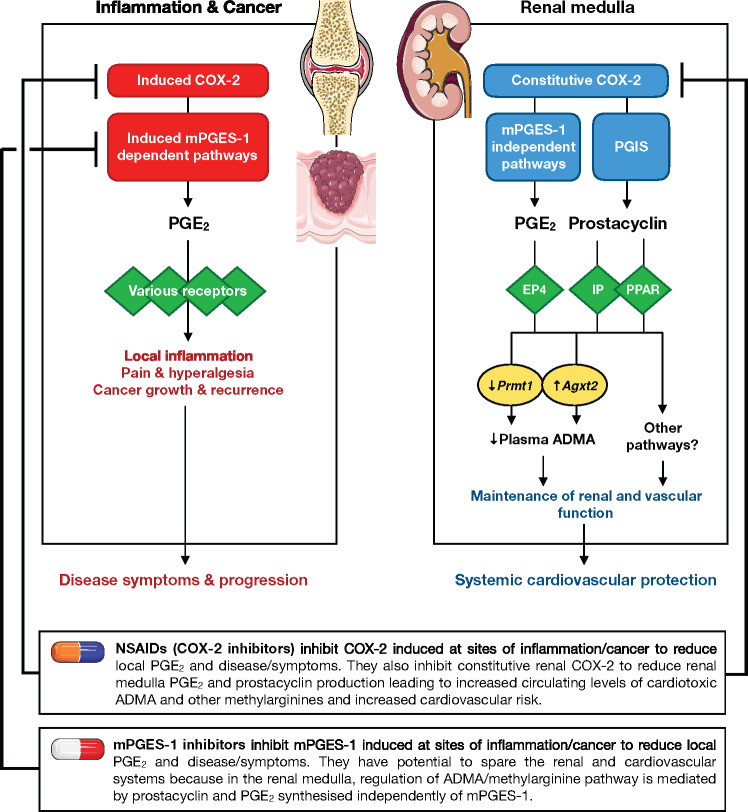

## 1. Introduction

Cyclooxygenase (COX) is a ubiquitous checkpoint in cardiovascular homeostasis and is present in two forms, COX-1 and COX-2.^1^ COX-1 is constitutively expressed throughout the body, including in platelets^2^ and endothelial cells,^3–5^ whilst COX-2 is restricted to specific regions^6^ which include the kidney^6–9^ where it is present in numerous cell types including fibroblasts, tubular epithelial cells, and endothelial cells.^9^ COX-1 in platelets drives prothrombotic thromboxane^4^^,^^10^ and is the therapeutic target of low-dose aspirin.^11^ In contrast, constitutively expressed COX-2 protects the cardiovascular system. We know this because mice lacking COX-2 are prone to atherosclerosis,^12–16^ thrombosis,^17–19^ and hypertension^8^^,^^19^ and because the non-steroidal anti-inflammatory drugs (NSAIDs) class of drugs, which all work by blocking COX-2-derived prostaglandin (PG)E_2_ and other prostanoids at the site of inflammation, cause much reported cardiovascular side effects. These side effects are associated with all members of the NSAID class except aspirin and increase personal risk of having a heart attack or stroke by as much as 30%^20–23^ even after only 2 weeks of regular use.^23^ Importantly, they amount to a global problem because NSAIDs are amongst the most commonly used pain medications worldwide and can prevent cancer. However, the precise mechanism(s) by which NSAIDs cause cardiovascular side effects are not completely understood causing serious consequences including that (i) there are no means of identifying patients at risk, (ii) NSAIDs are not used to prevent cancer, and (iii) the development of new drugs that target prostanoids has declined.

What we do know is that inhibition of protective prostanoids, particularly prostacyclin or PGE_2_, derived from constitutively expressed COX-2 in the kidney^8^ or at other sites,^19^^,^^24^ underpins cardiovascular toxicity of NSAIDs. With this in mind, selective drug targeting of PGE_2_ at the site of inflammation may well provide a therapeutic strategy to treat disease whilst sparing the release of cardioprotective prostanoids. This could be achieved by inhibition of microsomal prostaglandin (PG) E synthase-1 (mPGES-1), a prostaglandin synthase, which converts intermediates produced by COX-1 and COX-2 to proinflammatory PGE_2_.^25^ Inhibition of mPGES-1 is a well-developed area of pre-clinical research with studies showing that its genetic deletion protects against inflammation, pain and cancer,^26–28^ however, clinical development of mPGES-1 as a therapeutic target has been stopped. In some cases, this has been for specific reasons such as liver toxicity associated with LY3023703,^29^ but overall reflects a lack of a complete understanding surrounding NSAID cardiovascular toxicity and of relevant biomarkers.

Most recently work from our group^8^ and others^19^ has shown a link between inhibition of COX-2 and the endothelial nitric oxide synthase (eNOS) pathway which helps to explain how COX-2 protects the cardiovascular system. Our work additionally implicates the naturally occurring eNOS inhibitors asymmetric dimethylarginine (ADMA)^8^ and/or monomethylarginine (LNMMA)^8^ as biomarkers and mechanistic explanations of how loss of COX-2 mediates vascular dysfunction. ADMA is an established cardiovascular biomarker in both preclinical and clinical studies.^30^ However, the precise role of prostacyclin synthase (PGIS), mPGES-1, and associated downstream receptor signalling in the COX-2/ADMA axis is not known.

Thus, in the current study, we have used pharmacological tools and a full range of genetically modified mice to determine the precise involvement of COX-2, mPGES-1, PGIS, and respective prostanoid signalling receptors in the regulation of ADMA. This work validates and explains the ‘COX-2-ADMA axis’ and suggests an empirical estimation of the relative cardiovascular safety of mPGES-1 and COX-2 as therapeutic targets in man.

## 2. Methods

### 2.1 Animals

Male and female, 6- to 8-week-old wild-type mice, or mice lacking mPGES-1,^31^ PGIS (newly generated, see below), IP,^32^ PPARβ/δ,^33^ EP1,^34^ EP2,^35^ EP3,^36^ EP4,^37^ or DP1^38^ were used. Animals were housed in individually ventilated cages, with 12 h day/night cycle and free access to standard mouse chow and water. Studies were performed across multiple institutes, however in each case, (i) wherever possible samples were collected and data analysed by investigators blinded to the genotype/treatment of the animals, (ii) the same investigators collected and analysed tissue in all studies, (iii) tissue from relevant control animals was collect at the same time from the same source, and (iv) experiments were performed in accordance with all local guidelines, legislation and after ethical review and the Animals (Scientific Procedures) Act (1986) Amendment (2013). Experiments on mPGES-1^−^^/^^−^ mice, PGIS^−^^/^^−^ mice, and PPARβ/δ^−^^/^^−^ mice were performed at the Karolinska Institute, Sweden (approved by Karolinska Institute ethics committee, dnr. N86_13 and N364_11), Shantou Medical University, China (approved by the Shantou University Institutional Animal Research and Use Committee), and Nanyang Technological University, Singapore (approved by the Nanyang Technological University and SingHealth Institutional Animal Care and Use Committees in Singapore; IACUC SHS-868), respectively. For each of these lines corresponding wild-type littermates from the same colony were used as controls. Experiments on IP^−^^/^^−^, DP1^−^^/^^−^, EP1^−^^/^^−^, EP2^−^^/^^−^, EP3^−^^/^^−^, and EP4^−^^/^^−^ mice were performed at Goethe University, Germany (approved by the Animal Welfare Committee of the State Agency Darmstadt). These animals were maintained on a pure C57Bl/6 background and compared to age- and sex-matched C57Bl/6 mice from a separate colony held at the same institute. Experiments on IP^−^^/^^−^ and DP1^−^^/^^−^ were performed on separate occasions to those on EP1-4^−^^/^^−^ mice and therefore have their own individual control groups. All other experiments were performed at Imperial College London, UK (approved by the Imperial College Animal Welfare and Ethical Review Board under UK Home Office license 70/8422) on wild-type C57Bl/6 animals (Charles River, UK). Where indicated, wild-type mice were treated with the selective COX-2 inhibitor, parecoxib (100 mg/kg; Pfizer, USA) in drinking water for 5 days.^8^

### 2.2 Generation of PGIS^−^^/^^−^ mice

PGIS^−^^/^^−^ mice were generated by Beijing View Solid Biotechnology (Beijing, China) using transcription activator-like effector nuclease (TALEN).^39^ TALEN constructs targeting exon 2 of the *Ptgis* locus were designed by using TAL Effector Nucleotide Targeter 2.0 (https://tale-nt.cac.cornell.edu/ node/add/talen). The target sequences were: left 5′-GAGCCTCCGTTGGACCT-3′ and right 5′-CCAAGGCATGGCCCAGC-3′. All constructs were validated by DNA sequencing. TALEN mRNA was injected into mouse (C57BL/6) zygotes which were then transferred to pseudopregnant females to generate mutant founders (F_0_). Founders carrying frameshift mutations were intercrossed with wild-type mice to produce the F_1_ generation. PCR was performed with tail clip DNA from weaned mice with the primers: 5′-CAGCCTACTCTGACTTCCCCATG-3′ and 5′-GGGTGAGTGAAAGCGTATTTAATC-3′ for sense and antisense primers respectively. Mice were genotyped by sequencing the PCR products. The T7E1 (Beijing View Solid Biotechnology) assay was used to validate targeting efficiency and screen for the desired mutant mice. F_1_ mice with deletion of 14 bp (GCAGCATCCCCTGG) in exon 2 of the *Ptgis* locus were bred to produce PGIS^−^^/^^−^ mice.

### 2.3 Circulating mediators

Mice were killed by CO_2_ narcosis, blood collected from the inferior vena cava into heparin (10 U/mL final; Leo Laboratories, UK) and plasma separated. Levels of ADMA and arginine (DLD Diagnostika, Germany), the prostacyclin break-down product, 6-keto-PGF_1α_ (Cayman Chemical, USA), or creatinine (Cayman Chemical, USA) were measured by commercial biochemical/immunoassay kit.

### 2.4 Prostanoid release and measurement

Prostanoid release *ex vivo* was measured as we have previously described.^4^^,^^6^ Briefly, segments of renal medulla, renal cortex or aorta were incubated in DMEM media (Sigma, UK) containing Ca^2+^ ionophore A23187 (30 μmol/L; Sigma, UK) for 30 min at 37°C then release of PGE_2_ or 6-keto-PGF_1α_ was measured by immunoassay (Cisbio, France and Cayman Chemical, USA, respectively). In some cases, levels of a panel of eicosanoids was measured in the supernatant using an LC/MS/MS platform as previously described.^40^

### 2.5 Gene and protein expression

RNA was isolated from renal medulla and gene expression determined using TaqMan hydrolysis probes (Life Technologies, UK). Data were normalized to expression of the housekeeping genes 18S (probe ID: Mm03928990_g1) and *Gapdh* (probe ID: Mm99999915_g1) and relative expression compared using the comparative Ct method. Protein was isolated by homogenizing frozen tissue in PBS containing a protease inhibitor cocktail (Roche Bioscience, UK). mPGES-1 protein levels were measured using a specific ELISA (Mybiosource, USA) and normalized to total protein levels determined using the bicinchoninic acid method (Thermo Fisher Scientific, UK).

### 2.6 Statistics and data analysis

**Figure 1:**
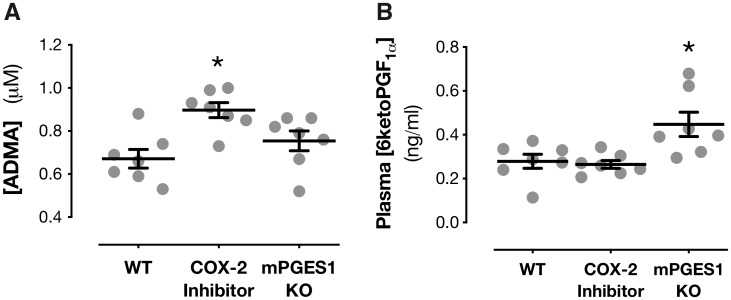
Effects of COX-2 inhibition and mPGES-1 gene deletion on plasma ADMA and 6-ketoPGF_1α_ in mice. Plasma levels of ADMA (*A*) or 6-ketoPGF_1α_ (*B*) in wild-type mice treated for 5 days with parecoxib (100 mg/kg/day p.o.) or mPGES-1 gene knockout (KO) mice. Data are mean ± S.E.M. (*A–B*): *P < 0.05 by one-way ANOVA with Dunnett’s *post hoc* test from *n* = 7 mice per group.

Data were analysed using Prism 7.0 software (Graphpad software, USA) and are presented as mean ± standard error for ‘*n*’ number of animals. *N* values for individual studies are given in figure legends. The experimental design for the primary endpoint of the study (plasma ADMA levels in mice where mPGES-1 was deleted or COX-2 was inhibited; *Figure 1A*) was based on formal power calculations. Effect size and variance were estimated from our previously published data on plasma ADMA in mice treated with parecoxib^8^ (Cohen’s *D* = 1.67) such that *n* = 7 provided 81% power by detect a significant difference (*P* < 0.05; two-tailed) in a three-group comparison. Subsequent mechanistic experiments were not the subject of formal power calculations. Differences were considered significant if *P* < 0.05. Details of statistical tests applied are given in each figure legend.

## 3. Results and discussion

### 3.1 Differential effects of mPGES-1 and COX-2 inhibition on plasma ADMA and prostacyclin

**Figure 2:**
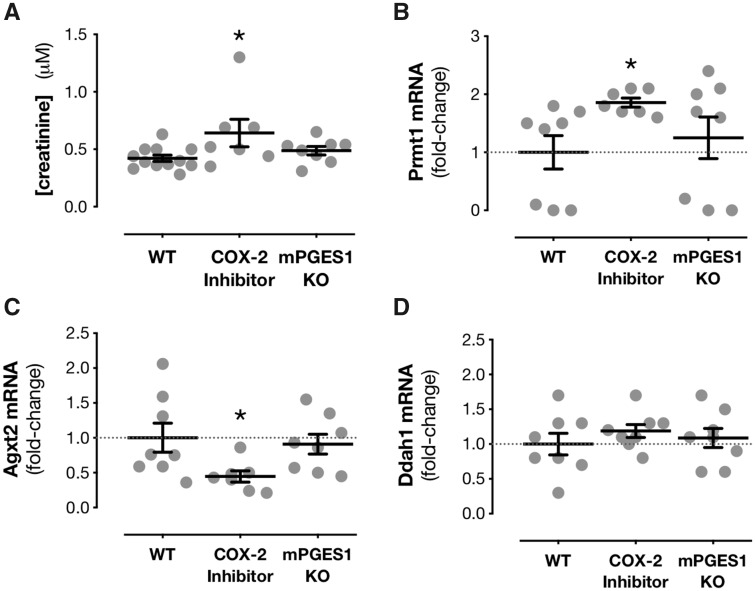
COX-2 but not mPGES-1 controls renal function and expression of methylarginine-related genes in the renal medulla. Plasma creatinine levels in wild-type mice treated for 5 days with parecoxib (100 mg/kg/day p.o.) or in mice where mPGES-1 has been knocked out (KO) (*A*). mRNA expression by qPCR of *Prmt1* (*B*), *Agxt2* (*C*), and *Ddah1* (*D*) in renal medulla of wild-type mice, wild-type mice treated with parecoxib or and mPGES-1 KO mice. Data are mean ± S.E.M. *P < 0.05 by one-way ANOVA with Dunnett’s *post hoc* test from *n* = 7–12 mice per group.

Two biomarkers have emerged as candidates to assess and predict the cardiovascular toxicity of anti-inflammatory drugs targeting COX-2 and/or the prostaglandin cascade. The first of these is prostacyclin, which is well established as a cardinal cardiovascular protective mediator derived from the COX pathway. Metabolites of prostacyclin can be measured in the urine, but these can be produced in the kidney^41^ and do not necessarily reflect prostacyclin production in the circulation.^4^^,^^42^^,^^43^ Instead, the prostacyclin breakdown product 6-keto-PGF_1α_ can be measured in the plasma, levels of which do correlate with prostacyclin production by systemic blood vessels.^4^^,^^6^^,^^44^ The second is ADMA, an established predictor of cardiovascular risk in the general population.^30^^,^^45^ Although to date no clinical data are available for the association between ADMA levels and cardiovascular risk in NSAID users, plasma ADMA is increased in COX-2 knockout mice with no associated change in plasma prostacyclin.^8^ Here, we show that ADMA is similarly increased plasma of wild-type mice where COX-2 is inhibited pharmacologically with chronic dosing (5 days) of parecoxib (*Figure 1A*). As with genetic deletion,^8^ COX-2 inhibition with parecoxib did not affect plasma levels of prostacyclin (*Figure 1B*). In contrast, loss of mPGES-1 had no effect on plasma ADMA but increased plasma prostacyclin (*Figure 1A* and *B*). We have previously shown that the increase in ADMA seen in COX-2 knockout mice is associated with renal dysfunction and mediated by changes in methylarginine-processing enzymes in the kidney.^8^ This point was recently corroborated in studies showing that ADMA was not increased and methylarginine genes not altered in models of reduced COX-2 that spare the kidney.^46^ In the current study plasma creatinine, a standard marker for predicting renal impairment, was increased in mice treated with parecoxib (*Figure 2A*) but unaffected in mPGES-1 knockout mice (*Figure 2A*). In line with this parecoxib increased expression of the gene encoding the ADMA synthetic enzyme PRMT1 (*Prmt1*; *Figure 2B*) and reduced expression of the gene encoding the ADMA metabolizing enzyme AGXT2 (*Agxt2*; *Figure 2C*). In contrast, deletion of mPGES-1 had no effect on *Prmt1* or *Agxt2* expression (*Figure 2*), which explains the lack of change in circulating ADMA levels (*Figure 1E*). Neither parecoxib treatment nor mPGES-1 deletion influenced expression of the gene encoding the alternative ADMA metabolic enzyme DDAH1 (*Ddah1*; *Figure 2D*). These observations suggest that, in direct contrast to COX-2 inhibition, targeting mPGES-1 spares both general renal function and the protective effects of renal COX-2 on the ADMA pathway. These findings agree with reports that mPGES-1 has a minimal role in the regulation of blood pressure and salt/water handling by the kidney in animal models^47^ and that in human healthy volunteers small, sporadic changes in plasma creatinine levels are not associated with changes in glomerular filtration rate or blood pressure.^48^ This further corroborates the idea that unlike COX-2, mPGES-1 does not play a substantial role in controlling cardio-renal physiology.^49^

With the data above demonstrating that mPGES-1 inhibition spares the renal COX-2/methylarginine pathway and boosts circulating prostacyclin levels, we went on to use these models to perform mechanistic investigations. We addressed the two underlying questions: (i) how and why do mPGES-1 inhibitors spare the COX-2/ADMA axis and (ii) how does mPGES-1 blockade boost vascular prostacyclin production? These are considered in turn below.

### 3.2 How does mPGES-1 blockade spare the COX-2/ADMA axis?

There are two possible scenarios that explain why loss of mPGES-1 does not result in increased ADMA. *Scenario 1*: PGE_2_ signalling does not regulate ADMA. *Scenario 2*: PGE_2_ does regulate ADMA but that mPGES-1 is not involved in PGE_2_ formation at the site where methylarginines are processed. To address scenario 1, we used a range of genetically modified mice where individual prostanoid genes were deleted and measured plasma levels of ADMA.

**Figure 3:**
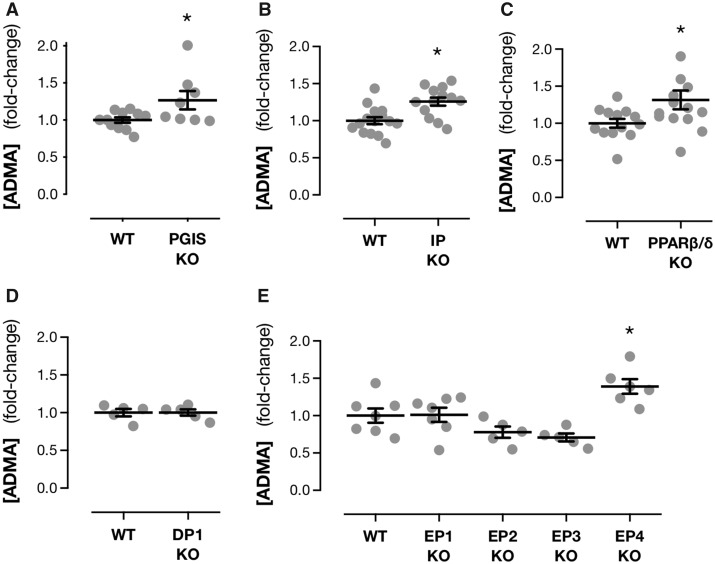
Both prostacyclin and PGE_2_ receptor signalling regulates plasma levels of ADMA. Plasma levels of ADMA in mice where prostacyclin synthase (*A*) or the prostacyclin receptors IP (*B*) or PPARβ/δ (*C*) or the PGE_2_/PGD_2_ receptors, DP1 (*D*) EP1, EP2, EP3, or EP4 (*E*) have been knocked out (KO). Data are mean ± S.E.M. for *n* = 5–15 mice in each group. Panel (*B*) includes *n* = 7–8 previously published values^8^ in addition to *n* = 6–7 new data points. *P < 0.05 by unpaired *t*-test (*A*–*D*) or one-way ANOVA with Dunnett’s *post hoc* test (*E*).

The role of PGE_2_ in the COX-2/ADMA axis has not been explored but there is evidence that implicates prostacyclin since we have previously reported that mice lacking the classical prostacyclin receptor, IP, have elevated plasma ADMA.^8^ However, in that study comparisons with other prostanoid pathways, including PGE_2_, were not made.^8^ Since prostacyclin may signal through other non-IP, prostanoid receptors, as well as nuclear receptors of the PPAR family,^50^ to fully evaluate the role of prostacyclin in controlling the renal ADMA axis, we generated a novel PGIS knockout mouse line where endogenous prostacyclin is completely removed and confirmed the predicted phenotype by measuring plasma 6-keto-PGF_1α_. Deletion of PGIS was associated with an almost complete lost of plasma prostacyclin (PGIS^+/+^, 400.6 ± 106.1 pg/mL; PGIS^−^^/^^−^, 29.0 ± 5.8 pg/mL; *P* = 0.002) and increased plasma ADMA (*Figure 3A*) to a similar degree as seen in mice treated with parecoxib (*Figure 1E*). To determine the signalling pathways downstream of prostacyclin generation responsible for ADMA regulation we studied mice lacking IP and PPARβ/δ. Deletion of either IP (*Figure 3B*) or PPARβ/δ (*Figure 3C*) resulted in elevation of plasma ADMA levels. However, deletion of the prostaglandin D_2_ receptor DP1, which can also be activated by prostacyclin and shares similar signalling to IP, had no effect on plasma ADMA levels (*Figure 3D*).

These data are entirely consistent with the idea that COX-2-derived prostacyclin production regulates ADMA levels but doesn’t however, rule out a similar or complementary functional role for COX-2/mPGES-1-derived PGE_2_. To address this possibility we studied ADMA levels in the plasma of a full range of PGE_2_ receptor knockout mice. PGE_2_ utilizes four classical receptors, EP1-4, each linked to distinct signalling cascades, with EP4 being associated with cardioprotective properties including vasodilation and inhibition of platelet aggregation.^1^ Plasma ADMA was unaffected by deletion of EP1, EP2 or EP3 (*Figure 3E*). However, plasma ADMA was increased in EP4 knockout mice (*Figure 3E*). These observations suggest that both prostacyclin and PGE_2_ exert breaks on plasma ADMA and thereby rule out scenario 1 as an explanation for why mPGES-1 blockade spares ADMA.

**Figure 4:**
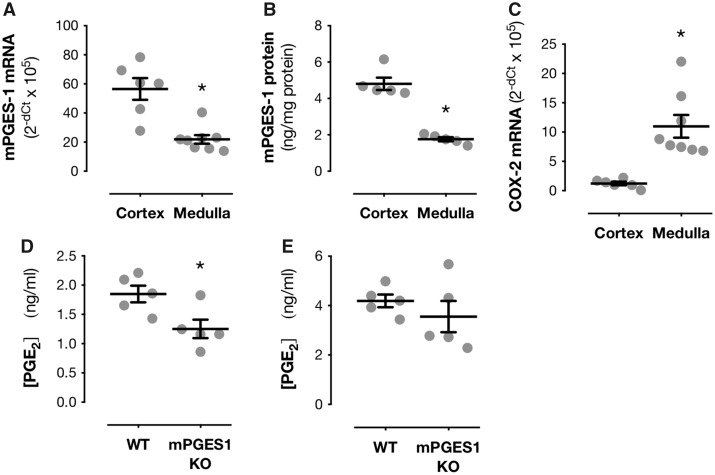
mPGES-1 and COX-2 have distinct compartmentalization within the kidney. Expression of mPGES-1 (*Ptges*) at mRNA level by qPCR (*A*) and protein level by ELISA (*B*) in renal cortex and medulla of wild-type mice. mRNA expression of COX-2 (*Ptgs2*) by qPCR in the renal cortex and renal medulla of wild-type mice (*C*). PGE_2_ production by isolated segments of renal cortex (*D*) and medulla (*E*) from wild-type and mPGES-1 knockout (KO) mice. Data are mean ± S.E.M. for *n* = 5–8 mice in each group. *P < 0.05 by unpaired *t*-test.

This leaves us with scenario 2; that mPGES-1 does not drive the ‘protective’ PGE_2_ which limits ADMA levels *in vivo*. We know that constitutive COX-2 and methylarginine pathways are co-localized specifically within the renal medulla and that here, rather than the cortex, or another site, is where NSAIDs act to increase ADMA. We know that both mPGES-1 and COX-2 are constitutively expressed in the kidney and that deletion of either gene reduces urinary markers of PGE_2_.^18^^,^^51^ Thus, to address scenario 2 we measured mPGES-1 expression and activity in the renal medulla and renal cortex. mPGES-1 was expressed at significantly higher levels in the renal cortex compared to the renal medulla at both the mRNA (*Figure 4A*) and protein level (*Figure 4B*) whilst, as we have previously shown, COX-2 was expressed almost exclusively within the renal medulla (*Figure 4C*). In line with this PGE_2_ levels in cortex from mPGES-1 knock out mice were reduced (*Figure 4D*) whilst levels in renal medulla were unchanged (*Figure 4E*). Levels of prostacyclin production by the renal cortex (wild type: 6.2 ± 0.7 ng/mL; mPGES-1^−^^/^^−^: 5.8 ± 1.1; *P* = 0.80; *n* = 5) or renal medulla (wild type: 14.2 ± 2.8 ng/mL; mPGES-1^−^^/^^−^: 19.2 ± 1.3; *P* = 0.13; *n* = 5) were not altered by deletion of mPGES-1 consistent with a specific effect on renal cortical PGE_2_ production. These observations show that mPGES-1 and COX-2 are oppositely compartmentalized within the kidney and explain why, despite PGE_2_ (via EP4) regulating ADMA, inhibiting mPGES-1 spares renal methylarginine processing. Although it would be advantageous to confirm this in human tissue such as biopsy material or cultured cells/organoids, such studies are limited by the rapid induction of COX-2 and mPGES-1 *ex vivo*.

### 3.3 How does mPGES-1 regulate prostacyclin production in vessels?

We next separately addressed the link between mPGES-1 deletion and vascular prostacyclin. The finding that deletion of mPGES-1 increases plasma prostacyclin is likely to reflect the well-recognized phenomenon that excess PGH_2_ substrate can be diverted between prostanoid synthetic pathways. This is in agreement with previous reports that urinary prostacyclin metabolites are increased in healthy volunteers receiving the mPGES-1 inhibitor, LY3023703.^48^ However, which tissues or cellular sites are involved in the shunting of PGH_2_ from mPGES-1 ⇒ PGIS are not known but important to consider since any drug which increases vascular prostacyclin has the potential to protect the cardiovascular system.

**Figure 5:**
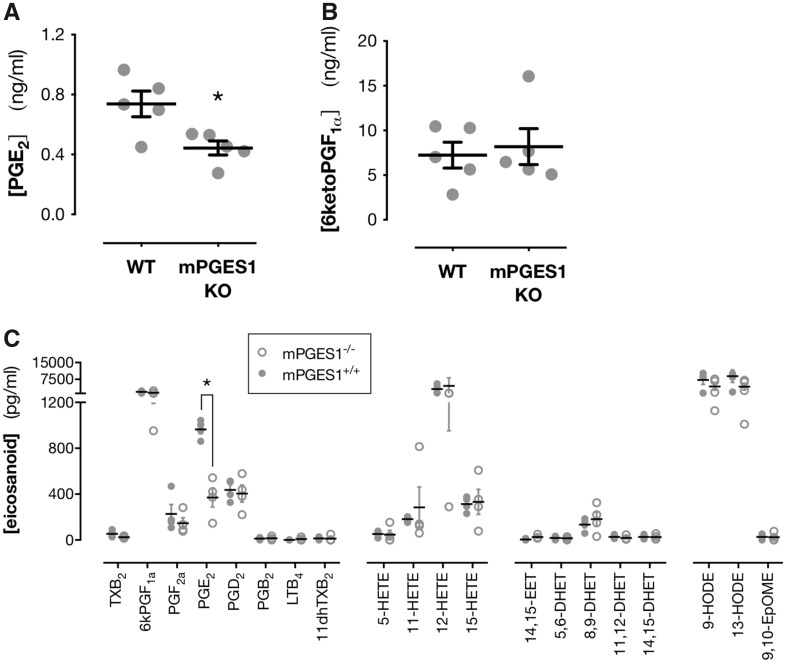
mPGES-1 contributes to constitutive vascular PGE_2_ production but its deletion does not increase local production of prostacyclin or other eicosanoids. Release of PGE_2_ (*A*) and 6-keto-PGF_1ɑ_ (stable breakdown product of prostacyclin) (*B*) and a full range of eicosanoids (*C*) by isolated aortic rings from wild-type and mPGES-1 knockout (KO) mice. For panel (*C*) only detectable eicosanoids are shown. The following mediators were assayed but were below limits of detection: PGH_2_, 8-iso-PGF_2a_, 8-iso-PGH_2_, 15-keto-PGE_2_, 20-OH-PGF_2a_, LTC_4_, LTD_4_, 20-carboxy-LTB_4_, 8-HETE, 19-HETE, 20-HETE, 20-HEPE, 11,12-EET, 8,9-EET, 17,18-DHET, 19,20-EpDPE, 12,13-EpOME, 12,13-DHOME, 9,10-DHOME, 19,20-DiHDPA, 17,18-EpETE, 22-HDoHE, AA, LA, 20-carboxy-AA. Data are mean ± S.E.M. (*A*, *B*): *P < 0.05 by (*A*, *B*) unpaired *t*-test for *n* = 5–7 mice per group. (*C*) Unpaired *t*-test with Benjamini–Hochberg FDR correction for *n* = 4 mice per group.

To understand the role that vascular PGIS plays in the shunting away from PGE_2_ towards prostacyclin when mPGES-1 is blocked we studied isolated aorta from wild-type and knockout mice. In blood vessels, studied immediately post-mortem to exclude any possibility of artefactual enzyme induction,^4^ prostacyclin (6-ketoPGF_1α_) was by far the most abundant prostanoid released, with levels ∼10 times higher than PGE_2_ (*Figure 5*). These observations are entirely consistent with what we know of vascular prostacyclin and PGE_2_ production.^1^^,^^4^^,^^13^ Nevertheless, release of PGE_2_ from freshly isolated aortic rings was reduced by mPGES-1 deletion (*Figure 5A*), suggesting mPGES-1 is constitutively expressed in large vessels where it contributes to physiological PGE_2_ production. However, we did not detect any concomitant increase in prostacyclin associated with reduced PGE_2_ production in the aorta (*Figure 5B*). Amongst other eicosanoids measured 12-HETE, 9-HODE, and 13-HODE dominated but were, as with prostacyclin, unaffected by mPGES-1 deletion (*Figure 5C*). These observations show that PGIS is expressed in excess in large blood vessels and that in this setting; the diversion of a small amount of PGH_2_ substrate from mPGES-1 does not impact on total prostacyclin levels within the vasculature. Our vascular results are limited to studies of the mouse aorta, however, together these observations suggest that mPGES-1 ⇒ PGIS shunting occurs in localized vascular beds or extra-vascular sites, the location of which remains the subject of investigation. Similarly, the potential for increased prostacyclin at those locations within the body to protect the cardiovascular (or other) systems has yet to be determined.

## 4. Conclusion

Our data show that in mouse models blocking mPGES-1 spares the COX-2/ADMA axis whilst increasing plasma prostacyclin levels. Further, mechanistic studies using mouse models suggest this can be explained by distinct compartmentalization of COX-2 and mPGES-1 in the kidney and the role of specific prostacyclin-sensitive receptors (IP, PPARβ/δ and EP4) in renal ADMA handling. However, prostanoid renal physiology and pharmacology can differ between species and validating our murine work in human tissue studies remains the subject of investigation. Nonetheless, this work reveals the downstream mechanisms that underpin the COX-2/ADMA axis (summarized in the graphical abstract) and emphasize the potential importance and added value of ADMA as a biomarker approach to assessing the cardiovascular safety of drugs that target the prostaglandin cascade.

## Authors’ contributions

Conceived/designed work (N.S.K., P.J.J., J.A.M.). Acquired/analysed/interpreted data (N.S.K., J.R., B.A., S.I.M., M.L.E., J.A.M.). Drafted manuscript (N.S.K., P.J.J., J.A.M.). Provided essential research tools/samples (B.L., M.L.E., M.G.C., X.W., W.W., D.C.Z., R.N., Y.Z.). Reviewed manuscript (N.S.K., J.R., B.A., B.L., S.I.M., M.L.E., M.G.C., X.W., W.W., D.C.Z., R.N., Y.Z., P.J.J., J.A.M.).

## Notes

Translational perspective Non-steroidal anti-inflammatory drugs (NSAIDs) treat pain and inflammation and can prevent cancer but cause serious cardiovascular side effects resulting in the virtual arrest in development of drugs which target prostanoid pathways. This includes new cyclooxygenase (COX)-2 blockers and inhibitors of microsomal prostaglandin E synthase-1 (mPGES-1). Our work has indicated that the well-established cardiotoxic biomarker, asymmetric dimethylarginine (ADMA), is increased when COX-2 is lost. In this study, we reveal the downstream signalling pathways responsible for the protective break that COX-2 exerts on ADMA and that this is independent of the mPGES-1 pathway. Together these studies, support the idea that ADMA has utility as a biomarker and that in this setting mPGES-1 inhibitors spare the pathways associated with NSAID cardiovascular toxicity.
